# Identification and functional characterization of a novel pathogenic *COL1A1* splicing variant in a Chinese family with osteogenesis imperfecta

**DOI:** 10.3389/fgene.2026.1758799

**Published:** 2026-02-06

**Authors:** Hongjuan Nie, Yuanxiong Chen, Peifang Qin, Guiming Xie, Di Zhang, Yifan Sun, Yongjun Mo

**Affiliations:** 1 Department of Clinical Laboratory, Eighth Affiliated Hospital of Guangxi Medical University, Guigang City People’s Hospital, Guigang, Guangxi, China; 2 Precision Diagnosis Center, Xiangya Third Hospital of Central South University, Changsha, Hunan, China; 3 Department of Hand and Foot Reconstructive Microsurgery, Eighth Affiliated Hospital of Guangxi Medical University, Guigang City People’s Hospital, Guigang, Guangxi, China

**Keywords:** COL1A1, minigene assays, osteogenesis imperfecta, splice, variant

## Abstract

**Background:**

Osteogenesis imperfecta (OI) is a hereditary disorder primarily caused by mutations in *COL1A1* or *COL1A2*, leading to bone fragility and deformities. Although numerous pathogenic variants have been identified, novel mutations in specific populations remain underreported, complicating diagnosis and genetic counseling.

**Methods:**

A Chinese family with mild type I OI was recruited. Whole-exome sequencing and Sanger sequencing were used to identify and validate a novel splice-site variant in *COL1A1*. Functional effects were assessed using two minigene constructs (pcMINI-*COL1A1* and pcMINI-N-*COL1A1*) transfected into HEK293T cells, followed by reverse transcription-polymerase chain reaction (RT-PCR) and sequencing of transcripts.

**Results:**

A novel heterozygous splice-site variant (c.298 + 1G>A) at the donor site of *COL1A1* intron 2 was identified and found to co-segregate with the disease. Minigene assays demonstrated that this mutation induces abnormal splicing patterns, including partial and complete skipping of exon 2, resulting in frameshifted transcripts with premature termination codons.

**Conclusion:**

The c.298 + 1G>A variant leads to aberrant splicing and likely haploinsufficiency, consistent with a mild OI phenotype. This study expands the *COL1A1* mutation spectrum and supports the use of functional assays for clarifying pathogenicity.

## Introduction

Osteogenesis Imperfecta (OI), commonly known as “brittle bone disease,” is a rare inherited connective tissue disorder affecting approximately 1 in 15,000 to 20,000 live births globally, primarily characterized by bone fragility, recurrent fractures, and skeletal deformities due to defects in type I collagen synthesis or processing (Marini et al., 2017). OI places significant burdens on patients, especially in the pediatric population where hospitalization rates are 8.4 times higher than the general population, and the majority of hospitalizations are due to fragility fractures (Storoni et al., 2022; Abraham et al., 2025). The disorder manifests a broad phenotypic spectrum, ranging from mild manifestations to perinatal lethality, and is classified according to the conventional Sillence system (types I–IV) (Forlino and Marini, 2016). Over 90% of OI cases stem from pathogenic variants in genes involved in collagen biosynthesis, predominantly *COL1A1* and *COL1A2*, though at least 18 other genes contributing to collagen modification, folding, or osteoblast function have been identified, with novel mutations continuously reported (Jovanovic and Marini, 2024; Yamaguti et al., 2023). This genetic complexity, compounded by population-specific mutation spectra and numerous undiscovered variants, hinders universal therapeutic strategies, necessitating ongoing exploration of new mutations.

*COL1A1* and *COL1A2* code for the α1 and α2 chains of type I procollagen, a key extracellular matrix protein in bone tissue. Pathogenic variants in these genes lead to OI through two principal mechanisms. The first involves quantitative defects, or haploinsufficiency, commonly caused by nonsense, frameshift, or splicing mutations that induce mRNA degradation and reduce normal collagen synthesis by about half; such mutations typically correlate with milder type I OI. The second mechanism entails qualitative defects characterized by dominant-negative effects. These often stem from missense mutations that replace glycine within the characteristic Gly-X-Y repeats, for example, with serine or aspartate, thereby disrupting triple-helix assembly and resulting in more severe OI forms, including types II through IV (Marini et al., 2017; Forlino and Marini, 2016; Jovanovic and Marini, 2024; Yamaguti et al., 2023). Recent studies highlight significant population-specific heterogeneity in OI genetics, as *COL1A1/COL1A2* mutation rates vary markedly between groups, reaching approximately 90% in Western (Zhytnik et al., 2017) and Japanese cohorts (Ohata et al., 2019) while being considerably lower in Indian (47.6%) (Selina et al., 2025) and sporadic Chinese cases (40%) (Ju et al., 2020). Ongoing research continues to identify novel and recurrent pathogenic variants, such as deep intronic and splice-site mutations that disrupt collagen processing, as well as helical glycine substitutions strongly associated with severe phenotypes (Jovanovic and Marini, 2024; Schouw et al., 2025).These variants have had clinical implications by facilitating definitive diagnosis and prognosis prediction with genetic testing in complex clinical scenarios. However, the ongoing discovery of novel mutations hinders personalized treatment and genetic counseling.

Although there is now knowledge of the pathogenic variants in the *COL1A1* gene, the challenge for clinicians still exists in classifying the pathogenicity of novel or rare variants (Jovanovic and Marini, 2024). The clinical geneticist cannot depend on the bioinformatics prediction tools to predict the effect of the variant since they are often inaccurate in predicting the effect on splicing and the protein. In addition, the genomic databases worldwide are biased towards the European population, resulting in a knowledge gap and bias of pathogenicity of variants that are found outside Europe. Hence, according to the American College of Medical Genetics and Genomics (ACMG) guidelines, these novel variants must be functionally validated, especially in a particular population (Richards et al., 2015).

In this study, we report the identification of a novel heterozygous *COL1A1* intron 2 splice donor variant, c.298 + 1G>A, in a Chinese OI cohort through whole-exome sequencing. This variant, which was absent from major population databases, was functionally characterized through reverse transcription-polymerase chain reaction (RT-PCR), revealing an aberrant transcript, and confirmed with a minigene splicing assay. This finding enriches the genetic and phenotypic spectrum of OI in the Chinese population, providing a crucial basis for precise molecular diagnosis and genetic counseling for individuals in underrepresented groups.

## Materials and methods

### Ethical approval

This study was approved by the Ethics Committee of the Eighth Clinical Medical College of Guangxi Medical University (E2025-003-01). Informed written consent was obtained from all participants involved in the study, ensuring that the ethical standards of research involving human subjects were upheld.

### Participants

A Chinese family diagnosed with OI was recruited for this study. The proband underwent extensive clinical examinations, physical assessments, and imaging studies, and provided medical records for evaluation. A definitive diagnosis of OI was established based on comprehensive medical records, clinical presentations, and genetic diagnosis conducted in the hospital.

### Whole exome sequencing (WES) and variant confirmation

WES was performed on a blood sample from the two affected individuals (II-2, III-1) and two healthy subjects (II-1, III-2). Initially, DNA was fragmented to create a sequencing library. Targeted gene exons and adjacent splice regions were captured and enriched using the Roche KAPA HyperExome chip. Variants were detected utilizing the MGISEQ-2000 sequencing platform. Stringent quality control standards were applied to the sequencing data, requiring an average sequencing depth of 200× for the targeted regions, with at least 98.5% of the sites having an average depth exceeding 20×. Sequencing reads were aligned to the UCSC hg19 human reference genome using BWA software, and duplicate sequences were removed. Base quality score recalibration was performed using GATK tools to identify single nucleotide variants (SNVs), insertion-deletion variants (INDELs), and genotyping. ExomeDepth software was utilized to detect copy number variations at the exon level. SNV annotations were performed using several databases, including the 1000 Genomes Project, ExAC, ESP6500, GnomAD, and a local BGI database. Variants of potential pathogenic significance were identified, and their classification was guided by the ACMG and AMP guidelines for sequence variant interpretation, with additional refinement from the ClinGen Sequence Variant Interpretation Working Group and the ACGS. The SIFT tool was used to evaluate the conservation of amino acid substitutions to assess the potential impact of the identified variants. Detected variants were subsequently confirmed through Sanger sequencing.

### RT-PCR analysis

In this study, the primers required for RT-PCR are shown in Table 1. The PCR amplification reaction system consisted of 12.5 µL of PrimeSTAR Max Premix (2× concentration), 0.5 µL of each primer (10 µM for both F and R), 1 µL of template DNA (100 ng/μL), and ddH_2_O adjusted to a final volume of 25 µL. The PCR amplification was performed using the following program, comprising 40 cycles: an initial denaturation at 98 °C for 3 min, followed by denaturation at 98 °C for 10 s, annealing at 60 °C for 5 s, extension at 72 °C for 20 s, and a final extension at 72 °C for 5 min. This method effectively amplified the *COL1A1* mutation, providing a foundation for subsequent splicing variant analysis.

**TABLE 1:** Clinical characteristics of the study family with OI.

Patient	Gender	Age (Years)	Height (cm)	Weight (kg)	Fracture times	Blue sclera	Hearing	Teeth	Vision
III-1	Female	11	130	25.0	3	Yes	Normal	Normal	Normal
II-2	Female	35	148	37.5	6	Yes	Normal	Normal	Normal
I-1	Female	70	140	42.5	7	Yes	Normal	Normal	Normal

### Prediction of splicing effects

To evaluate the potential impact of the identified variant on pre-mRNA splicing, the SpliceAI tool (https://spliceailookup.broadinstitute.org/) was utilized. This deep-learning-based tool predicts splice-altering variants by calculating delta scores ranging from 0 to 1, which represent the probability of a variant affecting splicing within a specified genomic window. Consistent with the standard cutoffs for SpliceAI, scores of 0.2 or higher (indicated in green) signify high recall, 0.5 or higher (yellow) are recommended for general use, and 0.8 or higher (red) denote high precision for predicting splicing defects. A delta score exceeding the 0.5 threshold is generally considered significantly deleterious.

### Cell culture

HEK293T cells were cultured in DMEM supplemented with 10% (v/v) fetal bovine serum (FBS), and 100 U/mL penicillin and 100 μg/mL streptomycin at 37 °C in a 5% CO_2_ atmosphere. Transfections were conducted following the manufacturer’s instructions using Polyetherimide (PEI) (PolyScience, Cat.No. 23966-2). Post-transfection incubation lasted for 24–48 h.

### Minigene construct design and analysis

Two Minigene plasmids were designed and constructed: pcMINI-*COL1A1*-wt/mut and pcMINI-N-*COL1A1*-wt/mut. The pcMINI-*COL1A1* plasmid included fragments of Intron 1 (270 bp), Exon 2 (195 bp), and part of Intron 2 (120 bp) inserted into the pcMINI vector, which comprised a universal ExonA-IntronA-multiple cloning site-IntronB-ExonB sequence. The pcMINI-N-*COL1A1* plasmid incorporated portions of Intron 1 (499 bp), Exon 2 (195 bp), Intron 2 (155 bp), and Exon 3 (35 bp) into the pcMINI-N vector. Specific primers (Supplementary Table S1) were employed to amplify the target fragments and introduce mutations, with PCR amplification of wild-type and mutant fragments performed using genomic DNA from normal individuals as templates. The amplified fragments were restriction-digested and ligated into the vectors before being transformed into DH5α competent cells. Single clones were selected for verification through colony PCR and Sanger sequencing to confirm successful Minigene construction. The constructed Minigene plasmids were then transiently transfected into the 293T cell line, following lipid-based transfection instructions, and samples were collected 24 h post-transfection. Total RNA was extracted from the cell samples according to kit instructions, and after determining the concentration, equal amounts of RNA were reverse transcribed to synthesize cDNA. PCR amplification utilized primers designed at both ends of the Minigene vector (pcMINI-F and pcMINI-R). The amplified products were assessed through agarose gel electrophoresis, and specific bands were excised and subjected to Sanger sequencing for confirmation of transcription.

## Results

### Clinical characteristics of the family

**FIGURE 1:**
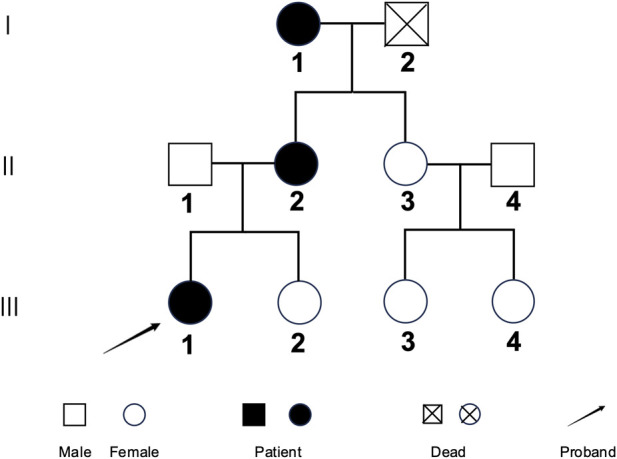
The pedigree of the family. Affected individuals are indicated by filled black, the proband is pointed out by an arrow.

The family analyzed in this study consists of three affected individuals and three unaffected relatives. Only the affected family members exhibited clinical features consistent with a diagnosis of type I OI. The pedigree of the family is presented in Figure 1, and their clinical characteristics are summarized in Table 1.

**FIGURE 2:**
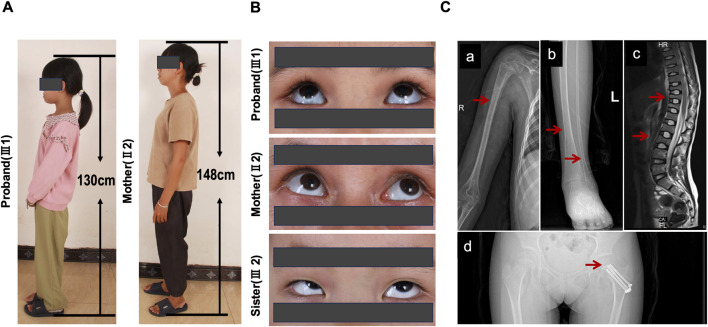
Clinical and radiological signs of the patients. **(A)** The heights of the proband and her mother. **(B)** The proband and her mother exhibited blue sclerae, whereas her sister did not. **(C)** Imaging findings in the proband. **(a)** Right humeral fracture. **(b)** Skeletal abnormalities of the left lower leg. **(c)** Compression deformities of thoracic and lumbar vertebrae. **(d)** Surgical internal fixation due to previous left femoral neck fracture.

The proband (III-1), an 11-year-old Chinese girl, has a height of 130 cm and a weight of 25 kg (Figure 2A). According to the standardized growth charts for Chinese children, the age- and sex-adjusted Z-scores for height and weight were calculated. The height Z-score was −2.27, indicating a lower growth status, while the weight Z-score was −1.56, corresponding to a medium-low growth status. Both her mother and grandmother also exhibit shorter stature and lower body weight compared to the general population (Figure 2A). All affected individuals presented with blue sclerae and a history of more than three fractures. Hearing, dentition, and vision were within normal limits. However, her sister did not have blue sclerae (Figure 2B). Imaging of the proband revealed a history of cumulative skeletal damage consistent with chronic bone fragility. X-rays of the right humerus showed evidence of a chronic, consolidated fracture (Figures 2A–C), while the left lower leg exhibited long-standing skeletal remodeling and cortical thinning (Figures 2B,C). These findings represent chronic, healed injuries rather than acute trauma. Furthermore, significant multiple vertebral compression deformities were identified in the thoracic (Th5-8, Th10-12) and lumbar (L1-3) vertebrae (Figure 2C). These deformities were characterized by classic biconcave ‘codfish’ appearances and varying degrees of vertebral height loss, indicative of chronic collapse due to long-term osteopenia. Additionally, Figures 2C,D displays consolidated internal fixation from a previous left femoral neck fracture, which has resulted in a stable limb-length discrepancy. The average lumbar spine bone density was measured at 113.180 mg/cm^3^, falling within the range of 80–120 mg/cm^3^. The patient was evaluated at our hospital for recurrent low-trauma fractures and thoracolumbar pain. The absence of blue sclerae and any fracture history in her six-year-old sister prompted further genetic testing and counseling to identify the underlying etiology. None of the affected family members had received bisphosphonate therapy or other relevant treatments.

### Sequencing analysis

Peripheral blood samples were collected from the family, including two affected individuals (II-2, III-1) and two healthy subjects (II-1, III-2). Following the proband’s request for genetic counseling and WES, bioinformatics analysis identified a novel *COL1A1* splicing variant, c.298 + 1G>A, which was validated through Sanger sequencing.

**FIGURE 3:**
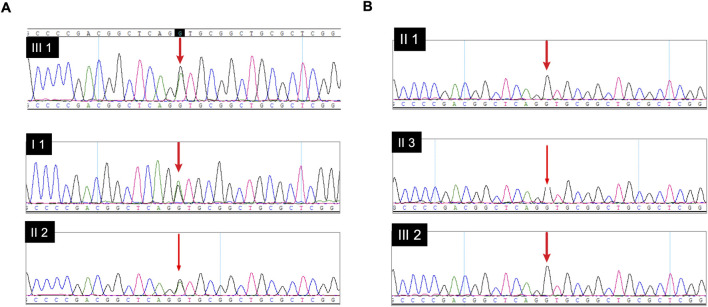
Sanger sequencing was performed to investigate the segregation of the *COL1A1* variant c.298 + 1G>A within family. **(A)** Sequencing result of affected individuals (I-1, II-2, and III-1). The red arrows point to the heterozygous “G>A” substitution at the splice donor site of intron 2. Note the overlapping peaks at the variant position, characteristic of a heterozygous mutation. **(B)** Sequencing results of unaffected relatives (II-1, II-3, and III-2), showing only a single “G” peak at the corresponding position. (Detailed raw sequencing data are available from the authors upon request).

To explore whether this variant segregated within the family, Sanger sequencing was performed on genomic DNA extracted from peripheral blood samples of the family. The results indicated that three affected individuals harbored the heterozygous *COL1A1* variant c.298 + 1G>A (I-1, II-2 and III-1, Figure 3A), in contrast to the three unaffected relatives (II-1, II-3 and III-2, Figure 3B). The variant was inherited by the proband (III-1) from her affected mother (II-2), who herself inherited it from her mother (I-1). The variant was absent in the proband’s younger sister (III-2) and in the older sister of individual II-2, consistent with the observed disease segregation in this family.

To evaluate the potential impact of the c.298 + 1G>A variant on mRNA splicing, we performed *in silico* analysis using the SpliceAI deep-learning algorithm (Supplementary Figure S1). The prediction results yielded a Donor Loss score of 1.00 at the position corresponding to the mutation (1 bp from the exon-intron boundary), indicating an extremely high probability of disrupting the original splice donor site. Additionally, a Donor Gain score of 0.66 was predicted at a position 34 bp downstream, suggesting the potential activation of a cryptic donor site. Both scores exceeded the recommended pathogenic threshold of 0.5, strongly supporting the hypothesis that the c.298 + 1G>A variant causes significant splicing aberrations at the donor site of intron 2.

### Splicing analysis of *COL1A1* c.298 + 1G>A in the minigene

**FIGURE 4:**
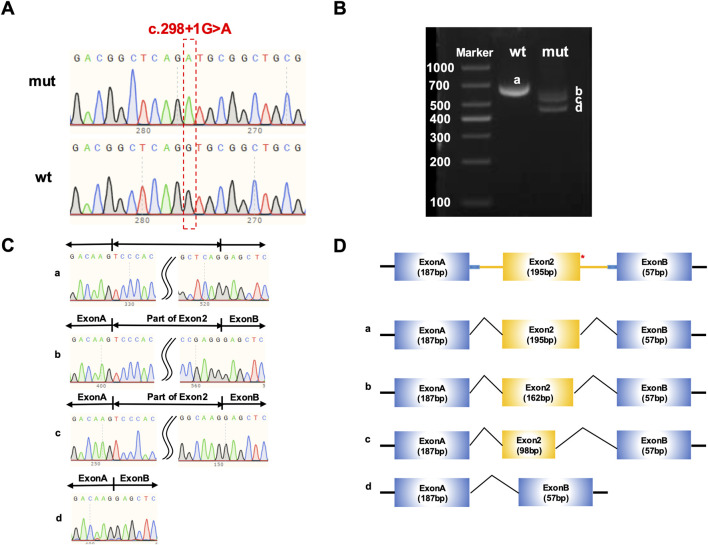
Functional analysis of *COL1A1* c.298 + 1G>A variant via minigene assay using pcMINI-*COL1A1* vector. **(A)** Sanger sequencing validation confirming successful insertion of wild-type and mutant COL1A1 minigenes into pcMINI-*COL1A1* vectors, ensuring construct integrity. **(B)** RT-PCR electrophoresis of HEK293T cells transfected with pcMINI-*COL1A1*-wild-type or mutant plasmids, displaying four distinct splicing product bands **(A–D)**. **(C)** Sanger sequencing verification of transcripts isolated from each band in **(B)**. **(D)** Schematic representation of splicing patterns: Wild-type lane: Exclusively produces band a, the canonical transcript (Exon A–Exon 2–Exon B). Mutant lane: Produces multiple aberrant transcripts, including band b (partial exon 2 skipping, 162 bp), band c (further shortened exon 2, 98 bp), and the most prominent band d (complete exon 2 skipping).

**FIGURE 5:**
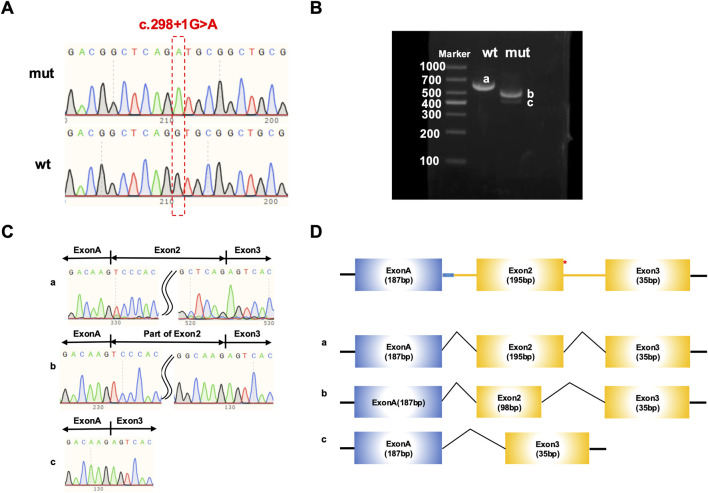
Functional analysis of *COL1A1* c.298 + 1G>A variant via minigene assay using pcMINI-N-*COL1A1*vector. **(A)** Sanger sequencing validation to confirm the successful insertion of wild - type and mutant *COL1A1* minigenes into pcMINI-N-*COL1A1* vectors, ensuring the correctness of vector construction. **(B)** Electrophoretic analysis of RT - PCR products from HEK293T cells transfected with pcMINI-N-*COL1A1*-wild-type or mutant constructs. Three distinct bands **(A–C)** are shown, representing different splicing products. **(C)** Sanger sequencing verification of the transcripts isolated from each band in **(B)**, which validates the accuracy of the identified splicing variants. **(D)** Schematic of the splicing events: Wild-type lane: Exclusively produces band a, the canonical transcript (Exon A–Exon 2–Exon 3). Mutant lane: Primarily produces band b (partial exon 2 skipping, 98 bp) as the dominant product, alongside band c (complete exon 2 skipping).

The minigene constructs for *COL1A1* c.298 + 1G>A were successfully validated through Sanger sequencing, as shown in Figures 4A, 5A. In HEK293T cells transfected with either pcMINI-*COL1A1*-wild-type or mutant constructs, electrophoretic analysis of RT-PCR products revealed four distinct bands (designated a–d, Figure 4B). Each corresponding transcript was isolated and validated by Sanger sequencing (Figure 4C). As shown in Figure 4D, the results indicated that band a corresponded to the canonical splicing product containing full-length Exon A (187 bp), Exon 2 (195 bp), and Exon B (57 bp). Band b exhibited partial exon 2 skipping, yielding a product consisting of Exon A (187 bp), a truncated Exon 2 (162 bp), and Exon B (57 bp). Band c represented a more extensive skipping event, with Exon A (187 bp), a further shortened Exon 2 (98 bp), and Exon B (57 bp). Finally, band d reflected complete skipping of Exon 2, resulting in a transcript comprising only Exon A (187 bp) directly joined to Exon B (57 bp). In the mutant (mut) lane of the pcMINI-COL1A1 construct, band d, which corresponds to the complete skipping of Exon 2, was identified as the most prominent product among the aberrant transcripts. This suggests that the total exclusion of Exon 2 is the predominant aberrant splicing event induced by the c.298 + 1G>A variant.

In HEK293T cells transfected with pcMINI-N-*COL1A1*-wild-type or mutant constructs, electrophoretic analysis of RT-PCR products yielded three distinct bands (a–c, Figure 5B). Each transcript was isolated and verified by Sanger sequencing (Figure 5C). As schematically summarized in Figure 5D, band a corresponded to the canonical splicing product, containing full-length Exon A (187 bp), Exon 2 (195 bp), and Exon 3 (57 bp). Band b displayed partial skipping of Exon 2, composed of Exon A (187 bp), a truncated segment of Exon 2 (98 bp), and Exon 3 (35 bp). Band c represented a more pronounced skipping event, resulting in a product consisting of Exon A (187 bp) directly linked to Exon 3 (35 bp), with complete exclusion of Exon 2. Regarding the pcMINI-N-COL1A1 construct, electrophoretic analysis revealed that band b (representing the partial skipping of Exon 2) appeared as the most intense band compared to the other mutant products. Despite the presence of multiple splicing patterns, the dominance of this frameshift-inducing transcript reinforces the likelihood of subsequent mRNA degradation.

## Discussion

In this study, we identified and functionally characterized a novel heterozygous splice-site variant (c.298 + 1G>A) in the *COL1A1* gene in a Chinese family with OI. By combining detailed clinical phenotyping, genetic sequencing, bioinformatic prediction, and minigene splicing assays, we provide strong evidence supporting its pathogenicity. This finding not only broadens the known mutational spectrum of *COL1A1* but also provides crucial insights into genotype–phenotype correlations in OI, which may facilitate more accurate diagnosis and targeted genetic counseling for affected individuals and their families.

In this Chinese family, classic clinical features of OI consistent with type I (mild) under the Sillence classification were observed. Key manifestations included recurrent fractures, blue sclerae, and the absence of severe skeletal deformities (Marini et al., 2017). These symptoms typically first appeared in infancy or childhood. While fracture frequency often decreases after adolescence, it may increase again during adulthood, particularly in postmenopausal women (Ralston and Gaston, 2020; Arshad and Bishop, 2021). The clinical presentation in this family aligns closely with the characteristics of mild OI as documented in the literature (Marini et al., 2017; Han et al., 2020; Huang et al., 2025), thereby excluding more severe forms such as type II (perinatal lethal) or type III (progressively deforming) OI.

Genetic analysis revealed that affected family members carried a heterozygous splice-site mutation in the *COL1A1* gene, which encodes the alpha1 chain of type I collagen—the primary structural protein in connective tissues such as bone, skin, tendons, and sclera (Dalgleish, 1997). Pathogenic mutations in *COL1A1* account for over 90% of all OI cases (Yamaguti et al., 2023; Zhytnik et al., 2017; Selina et al., 2025). Mutations in this gene are broadly categorized into two types: those leading to reduced collagen production (quantitative defects or haploinsufficiency) and those causing structurally abnormal collagen (qualitative defects or dominant-negative effects) (Jovanovic and Marini, 2024; Ju et al., 2020). Clinically, mild OI is commonly associated with quantitative defects, while severe or lethal OI often results from qualitative defects. The mild phenotype in this family strongly suggests that the novel c.298 + 1G>A splice-site mutation most likely leads to a quantitative defect. Specifically, we proposed that aberrant splicing triggered by this mutation results in transcripts that are degraded by nonsense-mediated mRNA decay (NMD), reducing the production of normal type I collagen.

To molecularly validate the pathogenic mechanism of the c.298 + 1G>A variant, a minigene splicing assay, which is considered the gold standard for functionally assessing splice-site variants, was employed (Anna and Monika, 2018). The experimental results strongly support the initial hypothesis. While the wild-type construct exhibited normal splicing patterns, the c.298 + 1G>A mutant led to the production of multiple aberrant transcripts. These abnormal splicing events, characterized by partial and complete exon skipping, disrupted the open reading frame and introduced a premature termination codon. Crucially, the most abundant transcripts identified in both minigene systems (band d in Figure 4 and band b in Figure 5) consistently introduced premature termination codons. Transcripts harboring premature termination codons are typically targeted for degradation via NMD, effectively preventing the synthesis of truncated protein from the mutant allele (Slayton et al., 2000; Willing et al., 1996). This “null allele” consequence provides a definitive molecular explanation for the mild OI phenotype observed in this family. By eliminating potentially deleterious α1 (I) chains, the NMD machinery prevents the accumulation of misfolded pro-collagen that would otherwise compromise the triple-helix assembly through a dominant-negative effect. Consequently, while the total output of type I collagen is reduced (haploinsufficiency), the molecules successfully secreted into the extracellular matrix remain structurally normal (Popp and Maquat, 2013). This quantitative deficiency leads to the characteristic bone fragility of Type I OI without the severe skeletal deformities associated with qualitative defects. These functional findings allow for the reclassification of the c.298 + 1G>A variant from a variant of uncertain significance (VUS) to a pathogenic mutation, establishing a robust genotype-phenotype correlation.

Although located in an intron, this mutation ultimately affects exon composition—a change particularly consequential in genes like *COL1A1*, where many exons encode Gly-X-Y repeats that form the triple-helical domain of type I collagen (Gensure et al., 2005; Pollitt et al., 2006). Notably, in-frame exon skipping—which does not cause a frameshift—can still disrupt the (Gly-X-Y)n repeats and impair triple-helix formation, often resulting in dominant-negative effects and severe phenotypes (Pollitt et al., 2006). However, both the minigene results and the mild clinical presentation in this family suggest that the c.298 + 1G>A mutation leads predominantly to frameshift-inducing splicing errors and subsequent NMD, rather than in-frame skipping. This supports the conclusion that the variant causes a quantitative rather than a qualitative collagen defect.

The findings of this study are consistent with previously reported *COL1A1* splice-site variants and add to the known mutational spectrum of the gene. For instance, several studies have documented that splice-site mutations in *COL1A1*—such as c.3814 + 1G>T reported by Han et al. (2020) and c.3531 + 1G>T identified by Huang et al. (2025)—frequently lead to aberrant splicing and are associated with mild OI type I, largely through mechanisms involving mRNA mis-splicing and nonsense-mediated decay (NMD), resulting in haploinsufficiency. Similarly, the c.298 + 1G>A variant described here induces exon skipping, corroborating the well-established pathogenic mechanism of quantitative collagen defects in mild OI. The specific location of this variant at the donor site of intron 2, which corresponds to an early region of the *COL1A1* gene, is particularly significant. Splice-site mutations in early exons, such as exons 1–5, that lead to NMD are hallmark causes of Type I OI. Our findings align with other early-region mutations where frameshift-inducing errors consistently result in mild phenotypes, in contrast to late-gene mutations that might bypass NMD and produce structurally defective collagen (Gökmen et al., 2024). This study identifies c.298 + 1G>A as a novel pathogenic variant in the Chinese population, further enriching the genotypic spectrum of OI. This mechanism aligns with the outcomes of numerous functional studies, including minigene assays performed in other cohorts, strengthening the claim that splice defects leading to in-frame disruptions and NMD are a common cause of type I OI. However, it is noteworthy that not all splice variants yield mild phenotypes; exceptions such as the deep intronic variant c.2451 + 77C>T reported by Schouw et al. (2025) can cause severe perinatal OI type II through in-frame insertions that disrupt the triple helix. The consistency between our functional results and those of previous studies underscores the utility of minigene assays in validating splicing defects and supports the reclassification of VUSs into pathogenic variants. Overall, this study reinforces the notion that splice-altering variants in *COL1A1* commonly operate through loss-of-function mechanisms and expand the genotypic and phenotypic correlations essential for diagnostic and counseling purposes.

Although this study provides compelling evidence, several limitations should be acknowledged. First, the analysis is based on a single family, which may limit the generalizability of the findings. Identification of the same variant in broader populations would strengthen the genetic evidence. Second, although the minigene assay in HEK293T cells accurately detected splicing defects, this *in vitro* model utilizes a non-bone-related cell line that may not fully replicate the specific splicing environment of osteoblasts. Furthermore, it could not directly evaluate the actual production and secretion of type I collagen in patient-derived cells. Future studies should analyze RNA and protein extracted from patient fibroblasts or osteoblasts to directly assess transcript abundance and collagen defects under physiological conditions, thereby providing more comprehensive mechanistic insights and verifying the *in vivo* relevance of our findings. Finally, while the identification of predominantly frameshifted transcripts supports a haploinsufficiency model, other potential pathogenic mechanisms, such as the potential for minimal protein products to escape nonsense-mediated decay, cannot be entirely excluded. Therefore, the generalizability of these conclusions should be interpreted with prudence, as the pathogenic process may be influenced by specific genetic or environmental modifiers within this family.

This work highlights potential therapeutic avenues rooted in gene-editing strategies. Emerging techniques, such as CRISPR/Cas9, offer promising opportunities for precise correction of disease-causing mutations (Jung et al., 2021; Cao et al., 2023). Future studies should explore the use of CRISPR/Cas9 to directly repair the c.298 + 1G>A variant in patient-derived cells or iPSCs, thereby restoring normal *COL1A1* splicing and collagen production. This approach may provide a curative strategy for individuals with this form of OI, moving beyond symptomatic management toward molecular correction (Chaugule et al., 2025; Deguchi et al., 2021).

## Conclusion

In summary, this study identified and functionally validated a novel splice-site variant (c.298 + 1G>A) in the *COL1A1* gene in a Chinese family with type I OI. Minigene assays demonstrated that this mutation disrupts canonical splicing, most likely leading to a quantitative defect in type I collagen production. These findings highlight the importance of integrating clinical phenotyping, genetic analysis, and functional studies for accurate diagnosis and pathogenic interpretation of variants in rare genetic disorders.

## Data Availability

Due to privacy concerns regarding the patients and their family members, the raw genetic data cannot be made publicly available. However, the specific variant data identified in this study are included in the article and Supplementary Materials. Further inquiries can be directed to the corresponding authors.
