# Supplementary material for: Identification and functional characterization of a novel pathogenic COL1A1 splicing variant in a Chinese family with osteogenesis imperfecta
Source: Front Genet. 2026 Feb 6;17:1758799. doi: 10.3389/fgene.2026.1758799 (PMC12919943; doi:10.3389/fgene.2026.1758799)
Supplement: Supplementary file 1 [file Table1.docx]

Supplementary Table 1. Primers employed in the construction and validation of the Minigene plasmid

| Primer | primer sequence（5′-3′） |
| --- | --- |
| pcMINI-COL1A1-BamHI-F | ATGGGTAGGTACCCGGATCCGGCTGAGGTTGGAGTTGGAA |
| COL1A1-mut-F | ACGGCTCAGATGCGGCTGCG |
| COL1A1-mut-R | CGCAGCCGCATCTGAGCCGT |
| pcMINI-COL1A1-EcoRI-R | GGGCAGTTTTCCTCGAATTCTTGCTAATGCTGCTCCCGTC |
| pcMINI-N-COL1A1-KpnI-F | ATGGCTCATGGGTAGGTACCAATCAGCCGCTCCCATTCTC |
| pcMINI-N-COL1A1-XbaI-R | GTTTAAACGGGCCCTCTAGACTCGACGCCGGTGGTTTCTT |
