# Supplementary figures and images for: Identification and functional characterization of a novel pathogenic COL1A1 splicing variant in a Chinese family with osteogenesis imperfecta
Source: Front Genet. 2026 Feb 6;17:1758799. doi: 10.3389/fgene.2026.1758799 (PMC12919943; doi:10.3389/fgene.2026.1758799)

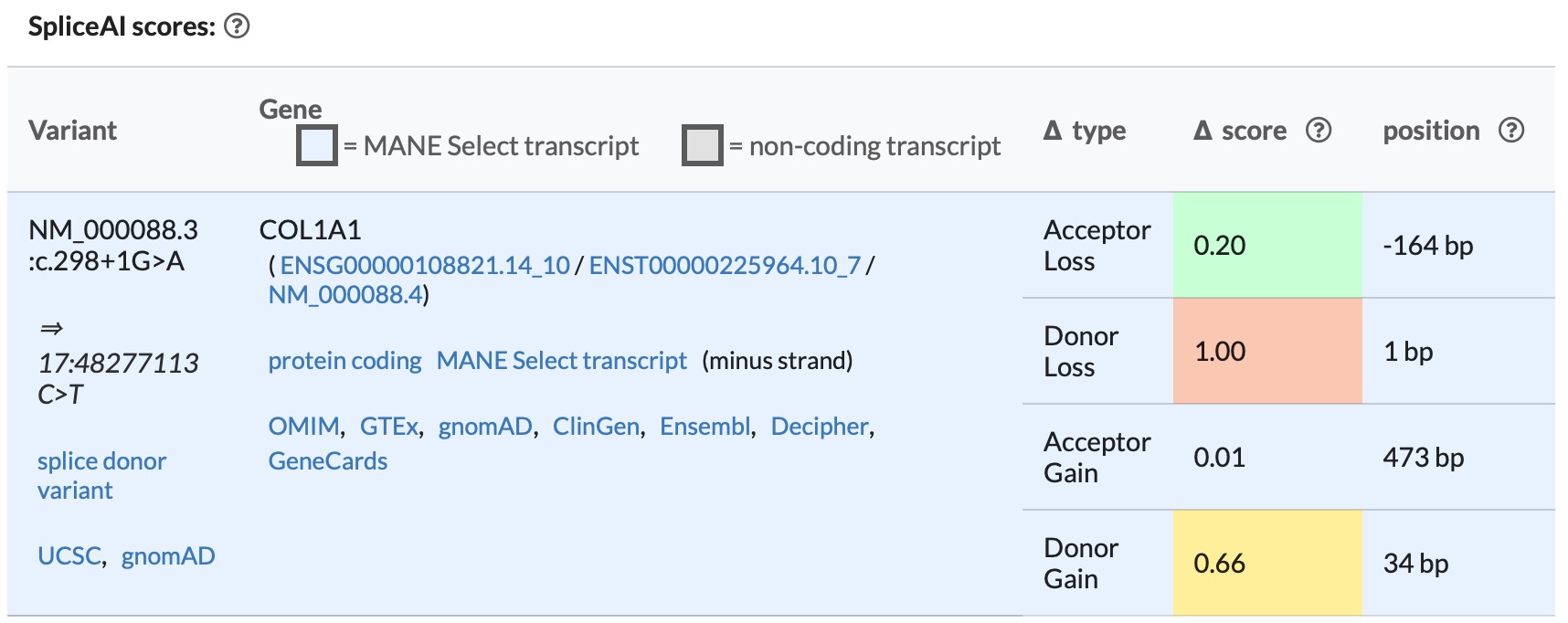

Supplement: Supplementary file 2 [file Image1.jpeg]
